# Cellular and extracellular proteomic profiling of paradoxical low-flow low-gradient aortic stenosis myocardium

**DOI:** 10.3389/fcvm.2024.1398114

**Published:** 2024-09-16

**Authors:** Manar Elkenani, Javier Barallobre-Barreiro, Moritz Schnelle, Belal A. Mohamed, Bo E. Beuthner, Christoph Friedemann Jacob, Niels B. Paul, Xiaoke Yin, Konstantinos Theofilatos, Andreas Fischer, Miriam Puls, Elisabeth M. Zeisberg, Ajay M. Shah, Manuel Mayr, Gerd Hasenfuß, Karl Toischer

**Affiliations:** ^1^ Clinic for Cardiology & Pneumology, University Medical Center Goettingen, Goettingen, Germany; ^2^ Department of Clinical Pathology, Faculty of Medicine, Mansoura University, Mansoura, Egypt; ^3^ Department of Biochemistry and Molecular Medicine, Medical School OWL, Bielefeld University, Bielefeld, Germany; ^4^ DZHK (German Centre for Cardiovascular Research), Partner Site, Goettingen, Germany; ^5^ King's College London British Heart Foundation Centre of Excellence, School of Cardiovascular Medicine & Sciences, London, United Kingdom; ^6^ Department of Clinical Chemistry, University Medical Center Goettingen, Goettingen, Germany; ^7^ Department of Medical Bioinformatics, University Medical Center Goettingen, Goettingen, Germany

**Keywords:** paradoxical low-flow low-gradient aortic stenosis, normal ejection fraction high-gradient aortic stenosis, myocardial biopsies, cellular and extracellular matrix proteomics, transcatheter aortic valve implantation (TAVI)

## Abstract

**Aims:**

Patients with severe aortic stenosis (AS), low transvalvular flow (LF) and low gradient (LG) with normal ejection fraction (EF)—are referred to as paradoxical LF-LG AS (PLF-LG). PLF-LG patients develop more advanced heart failure symptoms and have a worse prognosis than patients with normal EF and high-gradient AS (NEF-HG). Despite its clinical relevance, the mechanisms underlying PLF-LG are still poorly understood.

**Methods:**

Left ventricular (LV) myocardial biopsies of PLF-LG (*n* = 5) and NEF-HG patients (*n* = 6), obtained during transcatheter aortic valve implantation, were analyzed by LC-MS/MS after sequential extraction of cellular and extracellular matrix (ECM) proteins using a three-step extraction method. Proteomic data are available via ProteomeXchange with identifier PXD055391.

**Results:**

73 cellular proteins were differentially abundant between the 2 groups. Among these, a network of proteins related to muscle contraction and arrhythmogenic cardiomyopathy (e.g., cTnI, FKBP1A and CACNA2D1) was found in PLF-LG. Extracellularly, upregulated proteins in PLF-LG were related to ATP synthesis and oxidative phosphorylation (e.g., ATP5PF, COX5B and UQCRB). Interestingly, we observed a 1.3-fold increase in cyclophilin A (CyPA), proinflammatory cytokine, in the extracellular extracts of PLF-LG AS patients (*p* < 0.05). Consistently, immunohistochemical analysis confirmed its extracellular localization in PLF-LG AS LV sections along with an increase in its receptor, CD147, compared to the NEF-HG AS patients. Levels of core ECM proteins, namely collagens and proteoglycans, were comparable between groups.

**Conclusion:**

Our study pinpointed novel candidates and processes with potential relevance in the pathophysiology of PLF-LG. The role of CyPA in particular warrants further investigation.

## Graphical Abstract

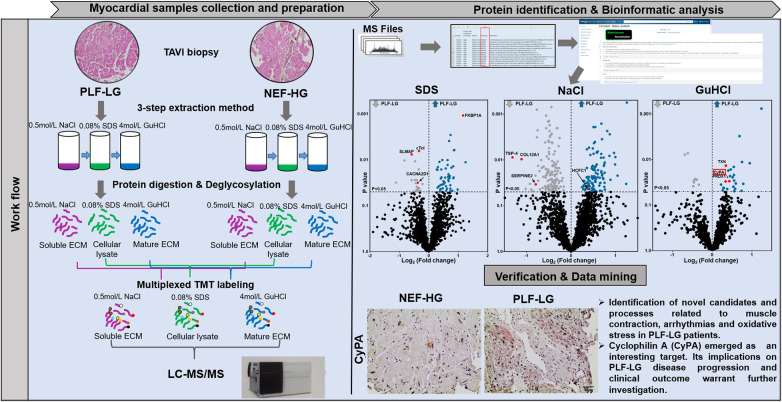

## Introduction

Paradoxical low-flow low-gradient aortic stenosis (PLF-LG) is a challenging clinical entity that affects approximately one-third of patients with degenerative severe aortic stenosis (AS) and appears to be more frequent in females and in older patients (1, 2). PLF-LG patients develop more advanced heart failure (HF) symptoms and have a lower survival rate than patients with normal ejection fraction, high-gradient AS (NEF-HG) (3, 4). Unlike NEF-HG, PLF-LG patients show contradicting echocardiographic parameters with a low mean aortic valve gradient (P_mean_) <40 mmHg despite the small aortic valve area (AVA) <1 cm^2^, and a low stroke volume index (SVI) ≤35 ml/m^2^ in the setting of a normal left ventricular (LV) ejection fraction (EF) ≥50%. This condition is mainly the result of pronounced LV concentric remodeling, a small LV cavity, myocardial fibrosis, elevated chamber stiffness and a restrictive filling pattern (5). In addition, the presence of other confounders such as uncontrolled systemic hypertension, mitral regurgitation and atrial fibrillation influence the low-flow state, resulting in a challenging assessment of the AS severity and potential delay of the appropriate intervention. This may negatively influence PLF-LG patient outcomes (6, 7). PLF-LG is also described as a form of valvular HF with preserved EF, i.e., HFpEF of AS, due to the high clinical and pathophysiological similarities between the two entities (8). The treatment options for all different hemodynamic subtypes of severe AS are generally limited to valve replacement—the current gold standard therapy—to mechanically unload the heart. With respect to PLF-LG, this therapeutic intervention is associated with a relatively poor clinical outcome as compared to other AS entities (9, 10), which underscores the continuing need to improve management strategies for these patients. For this, a better understanding of the molecular differences between hemodynamic subtypes of AS is essential. In recent years, major effort has been made to develop an extensive diagnostic workup using multiple diagnostic modalities and comprehensive clinical data interpretation for the accurate identification and diagnosis of PLF-LG (11, 12). Further research revealed that PLF-LG is not the end stage of NEF-HG but rather a separate hemodynamic entity characterized by progressive maladaptive LV remodeling (13).

To date, the molecular mechanisms driving PLF-LG are still poorly understood. This is to some extent due to the lack of available pre-clinical models as well as the difficulties involved in obtaining human myocardium samples. At the University Medical Center Goettingen (UMG), we have access to LV tissue samples from AS patients which were obtained during transcatheter aortic valve implantation (TAVI) (4). For the present study, we used these samples to analyze and compare the proteomic profile in LV myocardium of PLF-LG vs. NEF-HG, differentiating between the cellular and extracellular matrix (ECM) proteome via a three-step extraction method as recently described (14). The aim was to identify novel human targets and processes involved in the pathophysiology of PLF-LG with a distinct focus on the ECM proteins.

## Material and methods

### Patients

The study population included patients with severe AS who underwent transfemoral transcatheter aortic valve implantation (TAVI) at the University Medical Center Goettingen, as was recently described (4). Indication for TAVI was based on heart team consensus according to current guidelines (15). At baseline, transthoracic and transesophageal echocardiography (TTE and TEE), 6-min-walking test (6mwt), Minnesota Living with Heart failure Quality of life questionnaire (MLHFQ), New York Heart Association (NYHA) status and plasma N-terminal pro-brain natriuretic peptide (NT-proBNP) levels were recorded. Based on the current guidelines (15, 16) and as recently described (4), patients with an AVA ≤1.0 cm^2^, LV EF ≥50%, V_max_ ≥4 m/s or P_mean_ ≥40 mmHg were defined as NEF-HG, and those with an AVA ≤1.0 cm^2^ and indexed AVA ≤0.6 cm^2^/m^2^, LV EF ≥50%, V_max_ <4 m/s, P_mean_ <40 mmHg and a SVI ≤35 ml/m^2^ were categorized as PLF-LG. For this study, LV myocardial biopsies from 5 PLF-LG and 6 NEF HG patients with NYHA classes II and III were analyzed (Table 1).

**Table 1:** Patient characteristics.

	NEF-HG (*n* = 6)	PLF-LG (*n* = 5)	*P* value
Demographics			
Age (years)	83 ± 2.70	81 ± 3.30	0.562
Female, *n* (%)	5 (83.33%)	4 (80%)	>0.999
BMI (kg/m^2^)	28 ± 1.61	29 ± 1.15	0.548
Echocardiography			
LV EF (%)	60.98 ± 2.70	57.28 ± 4.36	0.473
SVI (ml/m²)	41.10 ± 2.37	26.64 ± 1.90	**0**.**001**
AVA (cm²)	0.71 ± 0.04	0.72 ± 0.06	0.965
Indexed AVA (cm²/m² BSA)	0.38 ± 0.02	0.37 ± 0.03	0.849
V_max_ (m/s)	4.37 ± 0.17	3.19 ± 0.14	**<0**.**001**
Peak gradient (mmHg)	46.17 ± 4.81	23.60 ± 2.48	**0**.**003**
LVMI (g/m² BSA)	151.1 ± 15.0	107.6 ± 14.7	0.070
IVS (mm)	17.83 ± 0.65	13.80 ± 0.58	**0**.**001**
LVEDD (mm)	41.00 ± 2.64	41.60 ± 3.14	0.886
Laboratory measures			
NT-proBNP (pg/ml)	4,203 ± 2,087	1,786 ± 619	0.389
Creatinine (mg/dl)	0.85 ± 0.08	1.10 ± 0.24	0.328
Medical history, *n* (%)			
Hypertension	6 (100%)	5 (100%)	>0.999
AF	2 (33.33%)	3 (60%)	0.567
CAD	3 (50%)	4 (80%)	0.545
Diabetes	2 (33.33%)	2 (40%)	>0.999
NYHA II	1 (16.66%)	0 (−)	>0.999
NYHA III	5 (83.33%)	5 (100%)	>0.999
Cardiac amyloidosis (Congo red staining)	0 (−)	0 (−)	–

Continuous variables are expressed as mean ± SEM, and categorical variables as numbers (percentages), Unpaired Student's *t*-test (for continuous variables) and Fisher's exact test (for categorical variables) were used for statistical analysis. BMI, body mass index; LV EF, left ventricular ejection fraction; SVI, stroke volume index; AVA, aortic valve area; BSA, body surface area; Vmax, aortic jet velocity; LVMI, left ventricular mass index; IVS, intraventricular septum; LVEDD, left ventricular end-diastolic diameter; NT-proBNP, N-terminal pro-B-type natriuretic peptide; AF, atrial fibrillation; CAD, coronary artery disease; NYHA, New York Heart Association.

Parameters with significant differences across groups are labelled in bold.

This investigation conforms with the principles outlined in the Declaration of Helsinki and was approved by the institutional ethics committee (approval number: 10/5/16). All patients provided written informed consent prior to participation in this study.

### Cardiac biopsy sampling

LV biopsies were performed as previously described (4). Briefly, they were obtained during TAVI from the basal anteroseptum using a biopsy forceps (Proflex-Bioptom 7 F, Medical Imaging Systems). One of five biopsies was fixed for 24 h in 4% paraformaldehyde (Roti® Histofix 4%, Carl Roth), washed with Dulbecco's Phosphate-Buffered Saline (Gibco, 14190-094) and fixed with paraffin for subsequent histological analyses. The other four biopsies were immediately preserved in liquid nitrogen and kept at −80°C.

### Proteomic profiling

For quantitative analyses of the cellular, newly synthesized and/or loosely bound ECM, and core ECM proteome, frozen cardiac biopsies from NEF-HG (*n* = 6) and PLF-LG (*n* = 5) were consecutively incubated with 0.5 mol/L sodium chloride (NaCl), 0.08% sodium dodecyl sulfate (SDS), and 4 mol/L guanidine hydrochloride (GuHCl) as previously described (14). Thus, from each cardiac biopsy we were able to isolate newly synthesized matrix proteins or loosely bound factors in the extracellular space (using 0.5 mol/L NaCl) before the tissues were decellularized (using 0.08% SDS) and integral ECM components were solubilized (using 4 mol/L GuHCl). Decellularization and ECM extraction yielded three extracts per sample: NaCl (enriched with newly synthesized and/or loosely bound ECM proteins), SDS (enriched with cellular proteins) and GuHCl (enriched with insoluble, highly integrated ECM proteins such as cross-linked collagens, proteoglycans and glycoproteins). Of note, due to the very small size of the obtained cardiac biopsies, protein extraction by NaCl and GuHCl was unsuccessful for one NEF-HG sample, resulting in an n-number of five (instead of six) for these analyses.

Digested samples from each extract were labeled with TMT10 plex Isobaric Mass Tag following the manufacturer's instructions (Thermo Scientific, 90406) and analyzed by liquid chromatography coupled with tandem mass spectrometry (LC-MS/MS). TMT labelling enables accurate quantification of complex protein mixtures, allowing assessment of expression changes across a wide dynamic range with excellent accuracy (17). A summary of the three-step extraction method and TMT labelling is illustrated in Supplementary Figure S1. Proteins were searched by Proteome Discoverer software. To define ECM and ECM-associated proteins in the NaCl and GuHCl extracts, the web platform “The Matrisome Project”, i.e., an open access database of core matrisome and matrisome-associated proteins (18), was utilized. As shown in Supplementary Figure S2A, the NaCl extracts were enriched with ECM/ECM-associated proteins, which were likely to be more soluble, in particular ECM-affiliated proteins and secretory factors. In contrast, the strongly bound ECM core proteins were predominant (68%) in the GuHCl extracts (Supplementary Figure S2B), confirming the successful overall coverage of the ECM components in the patient samples. A detailed method description is available in the online Supplementary File.

The mass spectrometry proteomics data included in this study have been deposited to the publicly available ProteomeXchange Consortium (19) through the PRIDE (20) partner repository with the dataset identifier PXD055391. Data visualization and quality assessment were performed using the PRIDE Inspector (21).

### Histological analysis of endomyocardial biopsies

Fixed biopsies were embedded in paraffin. Three μm paraffin sections were stained using Masson's trichrome (Sigma, HT15-1KT) for assessment of myocardial fibrosis or Alcian blue (Abcam, ab150662) to detect proteoglycans according to the manufacturers` instructions. Myocardial fibrosis was assessed by the same operator blinded to study groups and to clinical data using quantitative morphometry (Olympus Software cell-Sens 1.6). For immunohistochemistry, sections were deparaffinized and hydrated through graded series of ethanol. Antigen retrieval was performed by high temperature treatment with citrate buffer, pH = 6.0 (Dako, S2369) in a microwave. The slides were extensively washed with distilled water and the endogenous peroxidase was inhibited with 0.3% H2O2. Sections were rinsed with 1× PBS and then blocked with normal horse serum. Slides were incubated overnight at 4°C using either an anti-Cyclophilin A (Abcam, ab58144, 1:100), or an anti-EMMPRIN (Abcam, ab108308, 1:100) primary antibody. The following day, slides were rinsed in PBS and incubated in a horse anti-mouse/rabbit biotinylated IgG secondary antibody (VECTASTAIN Elite ABC Kit, Vector Laboratories, PK-6200). Sections were rinsed again, then incubated with an avidin/biotinylated enzyme complex (VECTASTAIN Elite ABC Kit, Vector Laboratories, PK-6200), rinsed again, and incubated with the 3-Amino-9-Ethylcarbazole (AEC) substrate chromogen (Sigma, 958D-30) for 5 min. The sections were then counterstained with hematoxylin (Vector Laboratories, H-3404), rinsed in tap water, and finally mounted in permanent mounting media. All sections were stained in the same histological sample run.

### Statistical analysis

Statistical analyses were performed with GraphPad Prism version 7.03 (GraphPad Software, Boston, USA). Two-tailed unpaired Student's *t*-test and Fisher's exact test were used where appropriate. All experiments were performed and analyzed in a blinded design, and data are presented as mean ± SEM unless otherwise noted. The criteria for identifying differentially abundant proteins (DAPs) between the investigated groups varied based on the type of extracts analyzed. DAPs were identified based on an adjusted *p*-value < 0.05, with fold changes ≥1.2 or ≤0.86 in the cellular extracts, and ≥1.1 or ≤0.9 in the ECM extracts (NaCl and GuHCl). Identification of ECM proteins in the NaCl and GuHCl extracts was done using a web platform (http://matrisomeproject.mit.edu/; last accessed on 17th July 2023) (18). Network analysis was constructed by STRING (https://string-db.org/; last accessed on 15th August 2023). Network visualization was carried out using the Cytoscape version 3.9.0. The protein-protein interaction network for the differentially abundant proteins was created utilizing the Cytoscape stringApp (22). Identification of network subclusters was performed using the Markov Clustering (MCL) algorithm implemented in the clusterMaker2 Cytoscape app (23). Functional overrepresentation analysis for the differentially abundant proteins was carried out using the R version 4.2.2. (https://www.R-project.org/; last accessed on 15th August 2023) and the enriched gene ontology (GO) function from clusterProfiler package version 4.2.2 (24). GraphPad Prism version 7.03 (GraphPad Software) was used to generate volcano plots.

## Results

### Patient characteristics

The demographics and clinical characteristics of patients included in the proteomic analysis are shown in Table 1.

### Cellular proteomic profiling in PLF-LG

**Figure 1:**
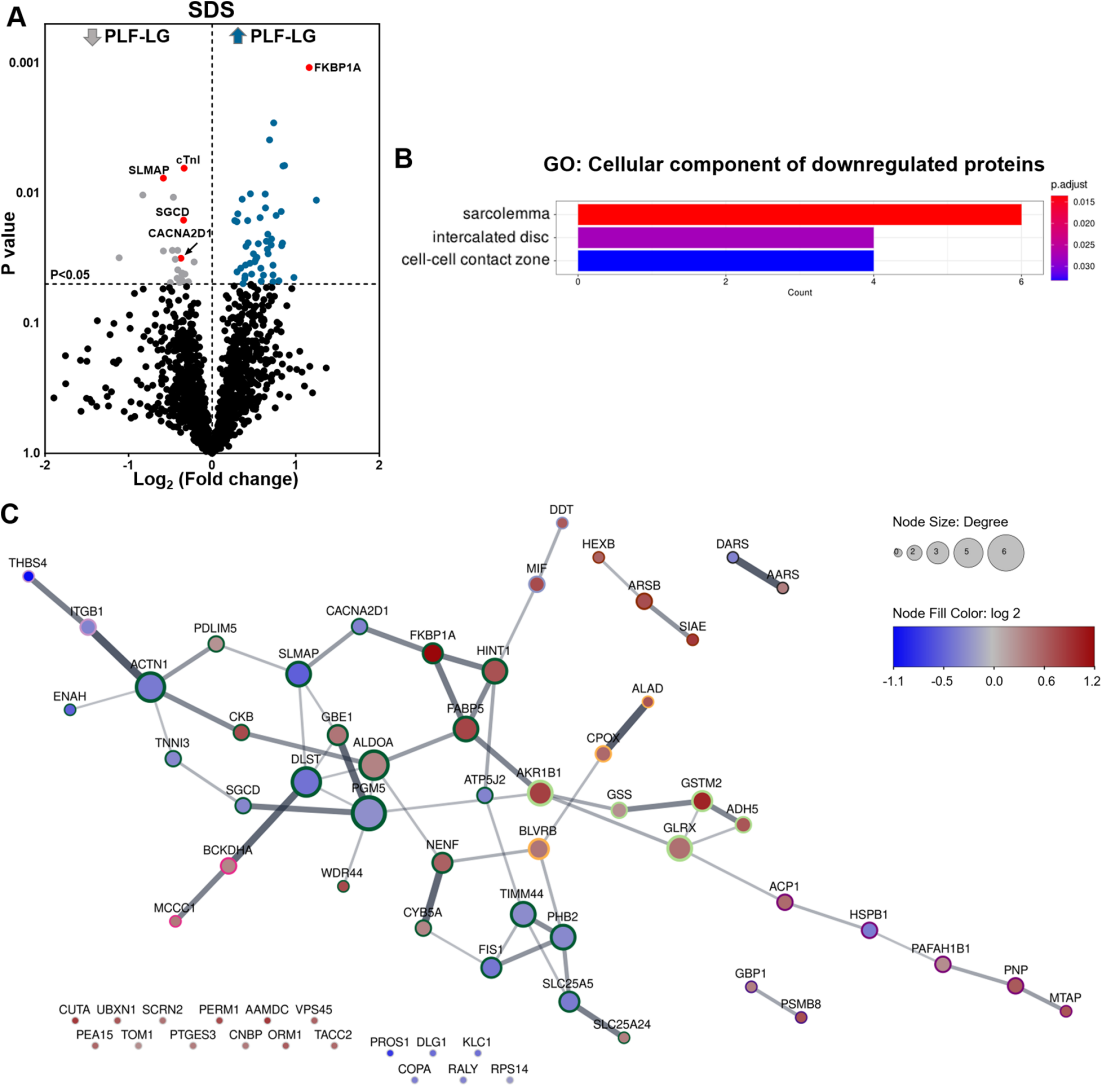
Proteomic profiling of cellular protein extracts from PLF-LG and NEF-HG patients. (**A)** Volcano plot of the quantified TMT labelled cellular proteins following SDS extraction. The *X*-axis represents the log_2_ fold change ratio (PLF-LG/NEF-HG) plotted against its significance level (*p* value). The blue and the grey dots represent the up- and downregulated proteins in PLF-LG vs. NEF-HG. *n* = 5 (PLF-LG) and 6 (NEF-HG). Proteins of interest with essential cardiac functions are additionally highlighted as red dots. **(B)** GO cellular component enrichment analysis of downregulated proteins in PLF-LG vs. NEF-HG. *Y*-axis represents the enriched GO terms, *X*-axis the number of downregulated DAPs. The color indicates the *p* value following Benjamini-Hochberg correction. **(C)** Protein-protein interaction network of differentially abundant proteins (DAPs) in PLF-LG vs. NEF-HG. Network nodes represent proteins and edges reflect physical and/or functional interactions of proteins. Node color reflects the protein abundance ratio (log_2_ fold change) ranging from low (blue) to high (red). Node size is proportional to node degree (i.e., number of edges adjacent to the node). Border color indicates cluster membership of each node.

Analysis of the cellular proteome in the SDS extracts yielded 1,689 proteins with at least two unique peptides per protein in each sample. By global statistical analysis, the quantified protein list was reduced to 73 differentially abundant proteins (DAPs) with a *p* value < 0.05 and a fold change cutoff of ≥1.2 increase or ≤0.86 decrease between the two groups. Of these DAPs, 50 proteins were upregulated and 23 proteins were downregulated in PLF-LG compared to NEF-HG (Supplementary Excel S1). The DAPs distribution, i.e., up- and downregulated in PLF-LG vs. NEF-HG, is presented as a volcano plot in Figure 1A. Subsequent GO analysis of downregulated proteins in PLF-LG demonstrated that these were mainly localized in the sarcolemma, intercalated disc and cell-cell contact zone (Figure 1B). In contrast, no GO terms were found to be enriched among the upregulated proteins. To gain further insight into the biological relevance of the DAPs, a network analysis was performed. About 68.5% of the DAPs were connected by direct or indirect interactions, forming ten major clusters. The largest cluster consisted of 24 protein nodes, including cardiac troponin I (cTnI), prolyl isomerase FKBP1A (FKBP1A), sarcolemmal membrane-associated protein (SLMAP), Delta-sarcoglycan (SGCD) and voltage-dependent Ca^2+^ channel subunit alpha-2/delta-1 (CACNA2D1), which suggests that processes such as muscle contraction, ion transport and calcium signaling are involved (Figure 1C).

### ECM proteomic profiling in PLF-LG

**Figure 2:**
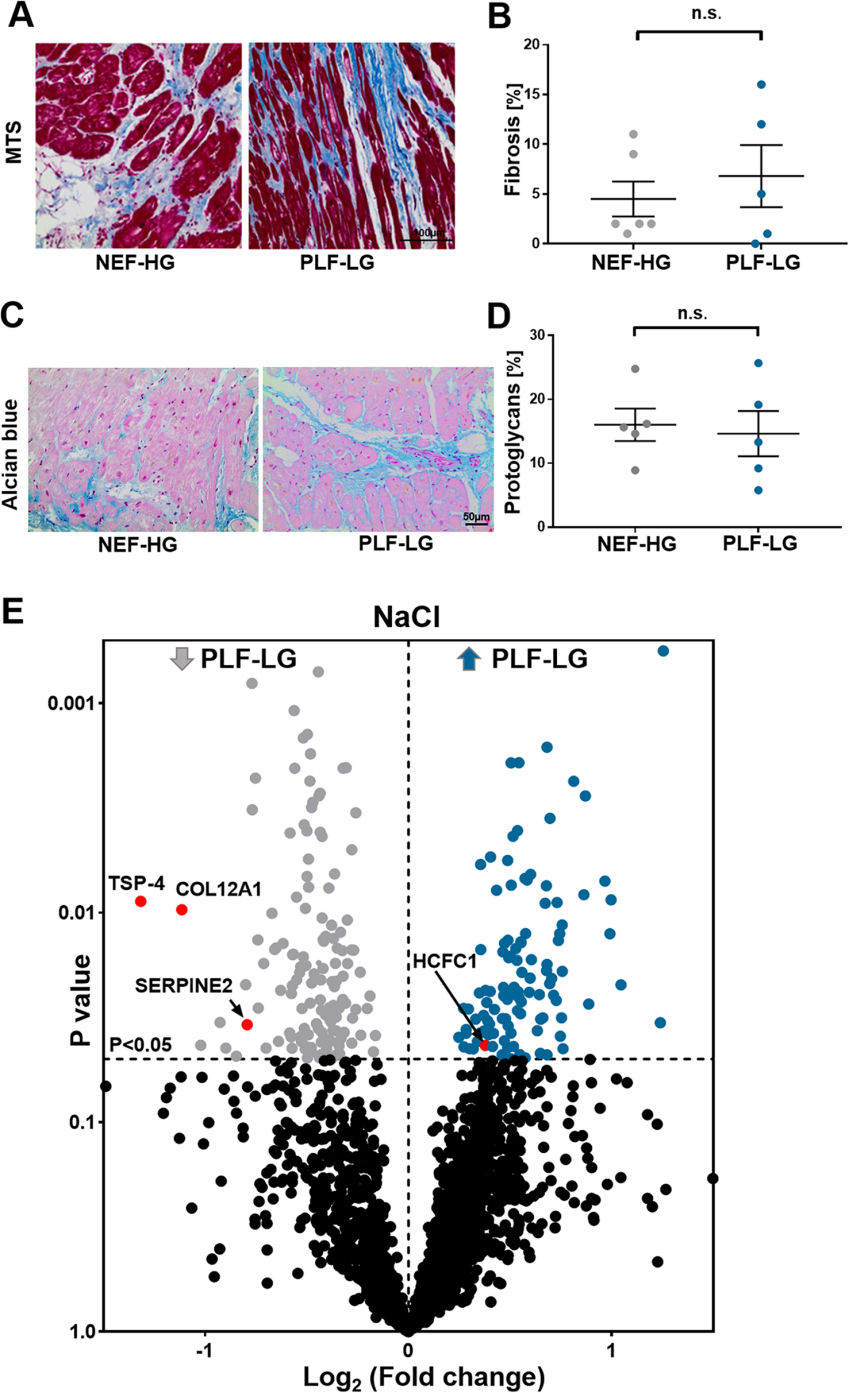
ECM proteomic profiling in LV biopsies from PLF-LG and NEF-HG patients. **(A)** Representative histological sections using Masson's trichrome staining (MTS) to assess fibrotic regions (blue). Scale bar: 100 μm. **(B)** Quantification of fibrotic area (% area). Data are presented as mean ± SEM, unpaired Student's *t*-test was used for statistical analysis, n. s., not significant. **(C)** Representative images of alcian blue staining showing proteoglycan accumulation (blue color). Scale bar: 50 μm. **(D)** Quantification of total accumulated proteoglycans (% area). Data are presented as mean ± SEM, unpaired Student's *t*-test was used for statistical analysis, n. s., not significant. **(E)** Volcano plots of the NaCl extraction. The *X*-axis represents the log_2_ fold change ratio (PLF-LG/NEF-HG) plotted against its significance level (*p* value). The blue and the grey dots represent the up- and the downregulated proteins in PLF-LG. *n* = 5 (PLF-LG) and 5 (NEF-HG). Proteins of interest with essential cardiac functions are additionally highlighted as red dots.

To evaluate the ECM content, cardiac biopsies were first stained for Masson's trichrome and alcian blue to quantify the level of ECM protein accumulation, in particular collagens (cardiac fibrosis) and proteoglycans, respectively. In line with previous studies (4, 25), no difference in total fibrosis between NEF-HG and PLF-LG could be detected (4.50 ± 1.765 vs. 6.80 ± 3.121; *p* = 0.519) (Figures 2A,B). The amount of accumulated proteoglycans was also comparable in both groups as shown by alcian blue staining (16.03 ± 2.542 vs. 14.63 ± 3.550; *p* = 0.757) (Figures 2C,D), indicating no major difference in the core abundant ECM content (collagens and proteoglycans) between the two groups.

Furthermore, a bioinformatic analysis and filtering of ECM extracts, i.e., obtained via NaCl and GuHCl, through MatrisomeDB (18), a comprehensive database platform for ECM-derived protein identification, were performed. Consistent with the histological analyses, differences in newly synthesized and/or loosely bound ECM proteins (i.e., NaCl-extracted proteins) and integral ECM proteins (i.e., GuHCl-extracted proteins) were less evident between the groups. Only four ECM/ECM-associated proteins were significantly altered in the NaCl extracts between the two groups: thrombospondin 4 (TSP-4), serpin peptidase inhibitor (SerpinE2), fibril-associated collagen with interrupted triple helices (FACIT) XII (COL12A1) and host cell factor 1 (HCF-1) (Figure 2E, Supplementary Table S1). Interestingly, none of the DAPs (*n* = 33) in the GuHCl extracts were identified as ECM/ECM-associated proteins (Supplementary Excel S2 and Table S2). Instead, abundances of the identified core ECM proteins in the GuHCl extracts [e.g., versican, biglycans, collagen alpha-2(V) chain, periostin, and fibrillin-1] were comparable between the groups (Supplementary Excel S2 and Table S2).

**Figure 3:**
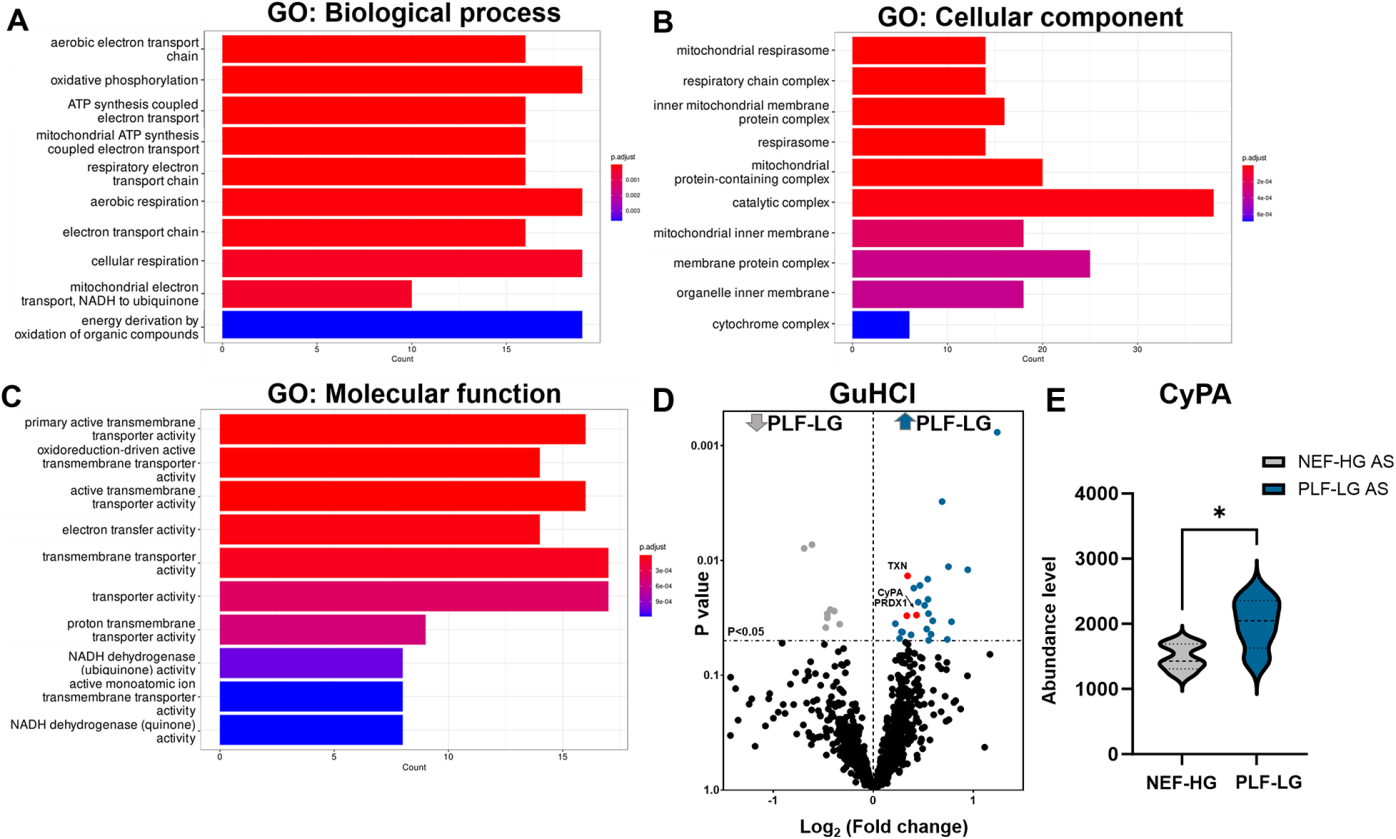
Analysis of non-ECM protein in the extracellular extracts (naCl and guHCl) in PLF-LG. Further analysis was performed in results from NaCl extracts. Bar graphs show the top ten most enriched GO terms of biological process category **(A)**, cellular component category **(B)**, and molecular function category **(C)** *Y*-axis represents the enriched GO terms; *X*-axis represents the number of downregulated DAPs in each term. The color indcates the *p* value following Benjamini-Hochberg correction. **(D)** Volcano plots of the GuHCl extraction. The *X*-axis represents the log_2_ fold change ratio (PLF-LG/NEF-HG) plotted against its significance level (*p* value). The blue and the grey dots represent the up- and the downregulated proteins in PLF-LG. Proteins of interest with essential cardiac functions are additionally highlighted as red dots. **(E)** Violin plot of CyPA abundance level in PLF-LG vs. NEF-HG. Dashed line indicates median; dotted lines indicate quartiles. Unpaired Student's *t*-test was used for statistical analysis; *p < 0.05 between the groups. *n* = 5 (PLF-LG) and 5 (NEF-HG).

Since the ECM also contains soluble proteins (e.g., growth factors and cytokines) that direct cellular recruitment and regulate gene transcription (26), we focused on identifying and quantifying non-ECM proteins that reside in the interstitial space and are important contributors to LV remodeling. Analyses of NaCl extracts revealed 238 non-ECM proteins with a significant variation in abundance between PLF-LG and NEF-HG (details of the complete list of significant altered proteins are provided in Supplementary Excel S3). Interestingly, GO enrichment analyses of upregulated proteins (*n* = 106) in PLF-LG patients could be related to mitochondrial function, including ATP synthesis, electron transport chain and oxidative phosphorylation (e.g., ATP5PF, COX5B, COX7C, NDUFS5, NDUFA8, FXN and UQCRB) (Figures 3A–C). No GO term enrichment was observed for the downregulated proteins (*n* = 132). With respect to the non-ECM proteins in the GuHCl extracts, only 33 DAPs between the groups were found as previously mentioned, resulting in a lack of GO term enrichment. Among these DAPs, antioxidant proteins such as thioredoxin (TXN), peroxiredoxin-1 (PRDX1) and the pro-inflammatory cytokine cyclophilin A (CyPA), were found to be upregulated in the GuHCl protein extracts of PLF-LG patients (Figure 3D).

### CyPA secretion in the ECM of PLF-LG

**Figure 4:**
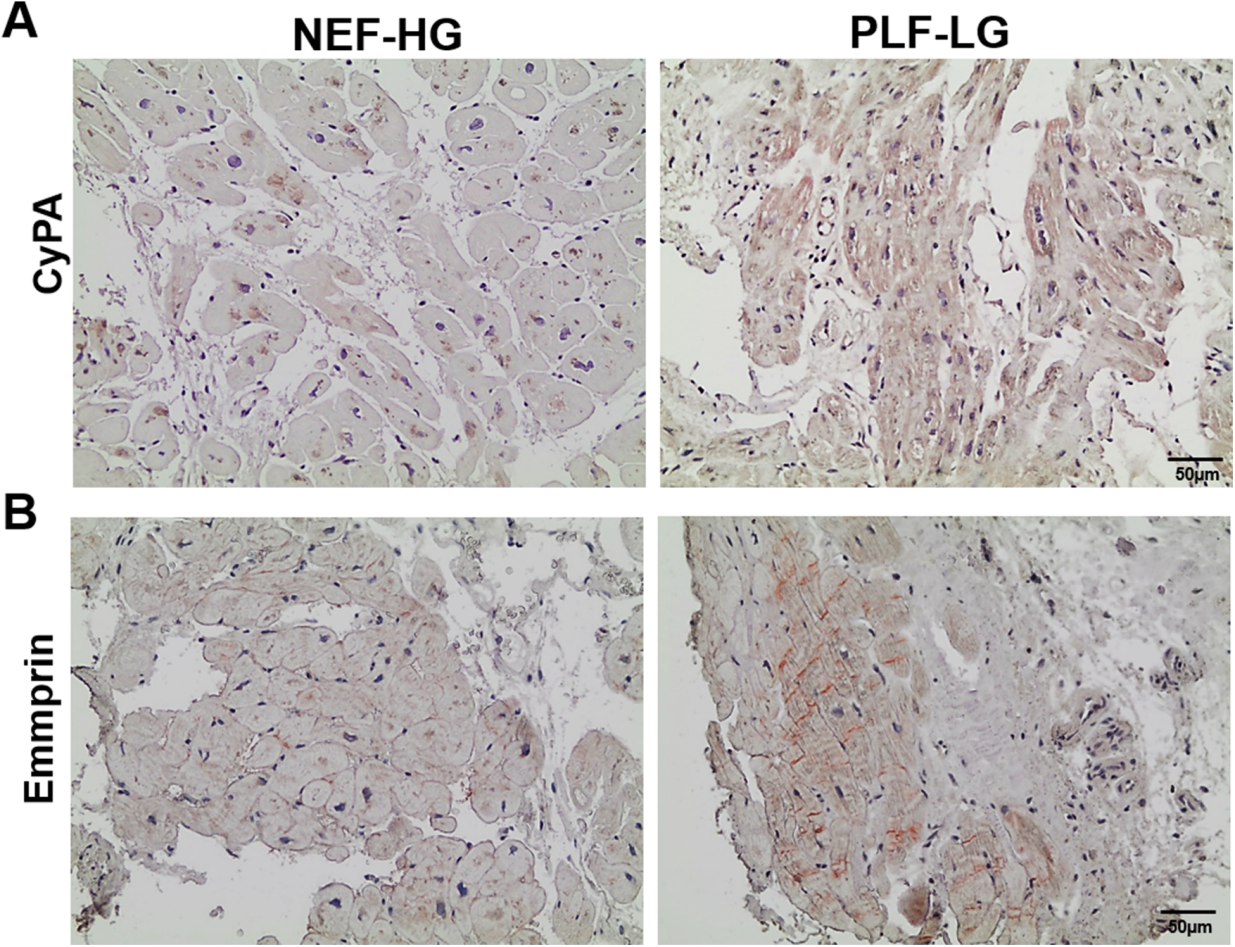
Subcellular myocardial localization of cyclophilin A (CyPA) and its receptor emmprin in PLF-LG and NEF-HG. Representative histological images showing LV myocardial sections immunostained for CyPA **(A)** and Emmprin **(B)** Positive immunolocalization for CyPA and Emmprin is indicated by brown staining, and histology/nuclear localization is indicated by blue-violet hematoxylin counter stain (*n* = 5/group).

Interestingly, we found that CyPA, an intracellular protein (27), was markedly upregulated in the GuHCl extracts of the PLF-LG patients (1.3-fold higher vs. NEF-HG, *p* = 0.03) as shown in Figure 3E), but no significant differences were detected in the SDS extracts (*p* = 0.69, Supplementary Excel S1), indicating that while the cytosolic CyPA levels remained comparable between the two groups, the extracellular CyPA protein levels were elevated in PLF-LG patients. To further investigate this, we performed immunohistochemical staining for CyPA in PLG-LG and NEF-HG LV sections. In line with the proteomic data, we found increased levels of CyPA in PLF-LG with a different distribution than in NEF-HG. In NEF-HG LV tissue, CyPA was found to be localized predominantly in the cytosol, but in PLF-LG samples accumulations of CyPA were diffuse at the cytosolic and the ECM levels (Figure 4A). We also evaluated the expression of Emmprin (or CD147), the extracellular receptor for CyPA. In line with our CyPA results, expression of Emmprin was also increased in the PLF-LG as compared to the NEF-HG samples (Figure 4B).

## Discussion

Distinct differences in the pattern of LV hypertrophy and cardiac dysfunction in response to different hemodynamic subtypes of AS have been previously described (4, 7), yet the differences in their molecular composition are still poorly understood. With respect to PLF-LG, the presence of unique clinical findings in the context of severe AS, low SVI despite a normal EF, indicate specific pathophysiological mechanisms. These need to be identified in order to develop disease paradigms and new therapeutic strategies for this particular pathological condition (28, 29). Due to the similar clinical presentation, it may be speculated that at least some of these mechanisms may also play important roles in diastolic HF, i.e., HFpEF (8).

In order to get a comprehensive perspective on the changes in myocardium proteome of patients with PLF-LG disease vs. NEF-HG, we combined an ECM enrichment procedure with a highly multiplexed quantitative MS approach. A major strength of this methodology is that it allows expanded coverage and resolution of ECM proteins as well as a high-throughput, deep proteome profiling of complex biological samples.

### Alterations in Ca^2+^ signaling proteins

Diastolic dysfunction is a prominent feature in PLF-LG patients and is predictive of all-cause mortality (30). Beside titin modifications and myocardial fibrosis, altered diastolic Ca^2+^ hemostasis is a key factor in development and progression of diastolic chamber stiffness and diastolic dysfunction. Cardiac troponin I (cTnI), the inhibitory subunit of the troponin complex, is a key regulatory protein in cardiac muscle contraction and relaxation. Experimental models with loss or mutations in cTnI have been associated with impaired relaxation and diastolic heart failure due to an increased myofibril sensitivity to calcium (31, 32). Alterations in sarcolemma proteins such as delta-sarcoglycan (SGCD), a member of the dystrophin-associated protein complex, also increase myofilament Ca^2+^ sensitivity, leading to diastolic dysfunction and cardiomyopathy (33, 34). Our proteomic analysis revealed a significant reduction of cTnI and SGCD levels in LV cellular extracts of PLF-LG vs. NEF-HG patients. These reductions may contribute to the diastolic impairment often observed in PLF-LG patients. In a larger study setting, it would be of great interest to assess whether the respective reductions of cTnI and/or SGCD protein levels may be associated with HFpEF development.

### Voltage-gated ion channels

The top abundant protein in the PLF-LG group was the prolyl isomerase FK506 binding protein (FKBP) 1A, also known as FKBP12.0, a member of FKBP family. It was previously shown that FKBP1A binds to cardiac ryanodine receptors (RyR2) and plays an important role in regulating its function (35). Enhanced expression of FKBP1A in isolated rabbit ventricular cardiomyocytes altered Ca^2+^ spark kinetics and increased sarcoplasmic reticulum (SR) Ca^2+^ content without affecting the Ca^2+^ transient (35). This finding is in contrast to the FKBP1B isoform (also known as FKBP12.6), which stabilizes the RyR2 and enhances the contractility upon overexpression (36, 37). In line with these findings, a recent study observed that mice with cardiomyocyte-restricted FKBP1A overexpression experienced enhanced arrhythmic propensity that resulted in sudden cardiac death (38). Paroxysmal atrial fibrillation (AF) was also documented in mice with cardiomyocyte-specific FKBP1A overexpression (39). Several studies have reported a high prevalence of AF in PLF-LG patients (2, 4, 40), so it is possible that FKBP1A is mechanistically involved.

SLMAP, one of the T-tubules and SR components, was among the top downregulated proteins in PLF-LG. *SLMAP* mutations have been described in Brugada syndrome (BrS), which results in impairment of the Nav1.5 trafficking, culminating in decreased peak *I*_Na_ density (41). Therefore, decreased SLMAP and increased FKBP1A may contribute to the arrhythmogenic phenotype seen in PLF-LG patients. Moreover, recent findings suggest that pathogenic mutations in *CACNA2D1* may mediate AF and contribute to BrS (42–44). Our data showed decreased CACNA2D1 in myocardial samples of PLF-LG patients, meaning that its loss might influence the arrhythmic risk in this group. Overall, these results indicate that PLF-LG patients exhibit profound dysregulation of ion homeostasis and are thus vulnerable to arrhythmias.

### ECM proteome

Our study showed less striking differences in the ECM proteome between the PLF-LG and the NEF-HG subtypes of AS. In the NaCl fractions, TSP-4 and Serpin E2 were differentially abundant between the investigated groups. TSP-4, a secreted anti-fibrotic ECM protein, was significantly downregulated in the PLF-LG patients. A reduction in TSP-4 has been shown to induce ECM deposition following cardiac pressure overload (45, 46). Similarly, SerpinE2 was also downregulated in our PLF-LG LV samples. SerpinE2 is a matrix remodeling protein that inhibits certain serine proteases which play important roles during the process of ECM degradation (47). The decreased TSP-4 and SerpinE2 in PLF-LG myocardium might contribute to the increased ventricular stiffness and the impaired diastolic function observed in those patients (5, 48).

The FACIT-type collagen 12A1 is thought to modulate organization and mechanical properties of collagen fibril bundles in tissues under biophysical stress (49). Host cell factor-1 (HCF-1) is a transcriptional co-regulator essential for basic cellular processes, including transcriptional regulation and cell cycle progression (50). Whether the differential decrease and increase of collagen 12A1 and HCF-1, respectively, as observed in this study, would affect the ECM integrity in PLF-LG hearts remains to be determined.

ECM protein accumulation, particularly the interstitial collagens, has been considered as a major determinant of tissue stiffness (51). In the GuHCl fractions, highly integrated ECM proteins such as collagens and proteoglygans were not altered between the PLF-LG and NEF-HG cases. This is in line with our histological analyses, which revealed comparable levels of LV fibrosis and proteoglycan accumulation. However, the functional impact of myocardial fibrosis extends beyond the mere quantity (i.e., severity of the deposition) to also include the quality (i.e., collagen phenotype shift and degree of cross-linking within collagen fibrils (52). In fact, tissue rich in collagen I is characterized by strength and stiffness, while those abundant in collagen III display enhanced elasticity. Furthermore, increased collagen cross-linking is likely to reduce tissue distensibility (53). In addition to cardiac fibrosis, post-translational modification of titin, a large sarcomeric protein, also plays a crucial role in determining myocardial passive stiffness. Gotzmann et al. reported titin-hypophosphorylation (at the elastic N2Bus domain, at residue S4185), albeit non-significantly, in the PLF-LG AS patients. This was associated with a significant shift in the titin isoform towards the more compliant N2BA variant; however, this shift might be a compensatory mechanism to counteract the increased myocardial stiffness resulting from the cardiac ﬁbrosis or titin-hypophosphorylation (25). Furthermore, acetylation of titin was recently reported to be associated with myocardial stiffness in HFpEF animal models (54) but there is limited knowledge regarding titin acetylation in PLF-LG AS patients. Additionally, we cannot rule out the possibility that increased diastolic Ca^2±^ may promote diastolic cross-bridge interactions, thereby contributing to an increase in diastolic stiffness (55). Ultimately, it is worth noting that the LV geometry, particularly the concentric hypertrophy observed in PLFLG patients itself may influence the diastolic stiffness (56). This gap in understanding highlights a potential area for further research into the molecular mechanisms affecting cardiac function in the PLF-LG AS group.

Given the minimal differences observed between the two groups in the core ECM proteins, our attention was redirected towards the less abundant ECM components and secreted proteins in the extracellular space. These are also important contributors to LV remodeling, but their precise roles are less explored. Since all ECM extracts were labelled with TMT, we were able to obtain quantitative accurate data for low abundant proteins that could be missed in label-free approaches (57). Indeed, we found accumulation of proteins related to oxidative stress in the PLF-LG biopsies vs. NEF-HG, as discussed below.

### Oxidative stress and CyPA

Oxidative stress is a key denominator in the pathophysiology of AS and directly promotes osteogenesis in valvular tissue (58, 59). In cardiomocytes, growing evidence suggests that oxidative stress and mitochondrial dysfunction can lead to the release of mitochondrial constituents into the extracellular milieu through mitochondrial extracellular vesicles (60, 61). In PLF-LG extracellular extracts, we found increased levels of mitochondrial proteins involved in oxidative phosphorylation and ATP synthesis, which may point to enhanced ROS production and oxidative stress. In line with this hypothesis, antioxidants such as thioredoxin and peroxiredoxin1 were significantly increased in PLF-LG extracellular extracts, indicating a compensatory response to the oxidative stress upon exposure to hemodynamic overload. Consistent with our findings, Brandenburg et al. reported significant upregulation of superoxide dismutase-2 (SOD2) and increased lipofusin deposits specifically in PLF-LG cardiac biopsies, which further points to oxidative stress as a fundamental process in the pathophysiology of this subgroup of AS (62).

Our proteomic results also showed selective upregulation of secreted (extracellular), but not cytosolic CypA in the myocardial extracts of PLF-LG patients. While several proteins in our study demonstrated altered levels in the extracellular extracts of PLF-LG patients, CyPA was particularly noteworthy due to its known cellular localization and the implications of its extracellular presence. CyPA is a highly conserved and ubiquitous protein that was initially believed to function primarily as an intracellular protein. More in-depth studies have revealed that it can be secreted by cells in response to inﬂammatory stimuli and oxidative stress. Mechanistically, extracellular CyPA (eCyPA) binds to its receptor EMMPRIN (also known as a cluster of differentiation, CD 147), thus promoting myofibroblast differentiation, fibrosis, cardiomyocytes hypertrophy, matrix metalloproteinase (MMP) activation and oxidative stress (63, 64). Clinical studies showed that plasma levels of CyPA are elevated in patients with HF, and values were found to be related to all-cause death and rehospitalization (65–67). Moreover, Zuern and colleagues reported that CyPA expression in myocardial biopsies is an independent predictor of high risk in patients with congestive HF (68). Additonally, CyPA knockout mice were protected from atherosclerosis (27) and angiotensin II–induced cardiac hypertrophy (69), and inhibition of CyPA expression in oxidative stress- and inflammation-related cardiovascular disorders attenuates the progression of the respective disease (69).

The consistent detection of CyPA in the extracellular heart extracts and its significant upregulation in the PLF-LG AS hearts, suggest that CyPA is actively released into the extracellular milieu of the PLF-LG hearts as a targeted systemic biological response rather than a general protein expression alteration, random variability, or an artifact of the experimental process. This makes CyPA not just another altered protein, but also a specific indicator of underlying pathophysiological processes such as inflammation or cellular stress response in PLF-LG AS. Thus, myocardial CyPA might serves as a potential therapeutic target for ameliorating the prognosis of the PLF-LG disease.

### Potential limitations

The present study is limited by the lack of non-failing hearts as controls, which could mask significant changes in the PLF-LG disease. The number of enrolled patients was also relatively small, which was due to the lack of available LV myocardial tissue material. The identified proteins thus need to be further verified in a larger patient cohort. Finally, this is an exploratory study that cannot conclusively identify causal relationships; however, our present data pave the way towards a better understanding of PLF-LG pathophysiology and provide a landscape proteome dataset that can be used by the research community for future hypothesis-driven research.

## Conclusion

Our comprehensive proteomic analysis uncovered a significant association between various proteins (e.g., cTnI, FKBP1A, CACNA2D1 and SLMAP) as well as processes (e.g., oxidative phosphorylation) and the PLF-LG disease. Additionally, the discovery of significantly elevated levels of myocardial CyPA in PLF-LG samples suggests its hitherto unknown role in the pathophysiology of this particular AS subtype. A more detailed mechanistic understanding of how these proteins may be involved in the pathophysiology of PLF-LG is thus essential for developing new therapeutic strategies to treat this pathological condition and improve HF symptoms.

## Data Availability

The mass spectrometry proteomics data presented in the study have been deposited to the ProteomeXchange Consortium via the PRIDE partner repository with the dataset identifier PXD055391 and 10.6019/PXD055391.
