# Supplementary material for: Cellular and extracellular proteomic profiling of paradoxical low-flow low-gradient aortic stenosis myocardium
Source: Front Cardiovasc Med. 2024 Sep 16;11:1398114. doi: 10.3389/fcvm.2024.1398114 (PMC11443424; doi:10.3389/fcvm.2024.1398114)
Supplement: Supplementary file 4 [file Datasheet1.docx]

**SUPPLEMENTARY MATERIAL**

**Cellular and extracellular proteomic profiling of paradoxical low-flow low-gradient aortic stenosis myocardium**

***Short title: Proteomics in paradoxical LF-LG aortic stenosis***

**Manar Elkenani, Javier Barallobre-Barreiro, Moritz Schnelle, Belal A. Mohamed, Bo E. Beuthner, Christoph F. Jacob, Niels B. Paul, Xiaoke Yin, Konstantinos Theofilatos, Andreas Fischer, Miriam Puls, Elisabeth M. Zeisberg, Ajay M. Shah, Manuel Mayr, Gerd Hasenfuß, Karl Toischer**

**Correspondence:** [manar.el-kenani@med.uni-goettingen.de](mailto:manar.el-kenani@med.uni-goettingen.de) & [ktoischer@med.uni-goettingen.de](mailto:ktoischer@med.uni-goettingen.de)

**Detailed Methods**

**Tissue extraction for proteomics**

About 10-20 mg of cardiac tissue were used for the sequential "three-step extraction method". Biopsies were incubated in sodium chloride (0.5 mol/L NaCl, 10 mmol/L Tris, pH=7.5, 25 mmol/L EDTA, supplemented with a protease inhibitor cocktail from Sigma, P8340) buffer (10:1 buffer volume to tissue weight) with slow agitation for 4 h. Supernatants were removed and stored at -80 °C until use. The tissues were then decellularized by incubation in a sodium dodecyl sulphate (SDS) (0.08% SDS, 25 mmol/L EDTA, supplemented with proteinase inhibitor cocktail) buffer (10:1 buffer volume to tissue weight) and incubated at room temperature (RT) for 16 h with continuous mixing at low speed. Afterwards, the SDS fraction was transferred into a new tube and kept at -80 °C for later use. Tissue pellets were finally incubated in guanidine hydrochloride buffer (4 mol/L GuHCl, 50 mmol/L sodium acetate, pH=5.8, 25 mmol/L EDTA supplemented with protease inhibitor cocktail) buffer (5:1 buffer volume to tissue weight) at RT and vortexed vigorously for 72 h to enhance mechanical disruption of the ECM components. Supernatants were centrifuged at 16.000 × g for 10 min at 4 °C and stored at −80 °C until use.

Twenty µg proteins of ECM extracts (NaCl and GuHCl) were further precipitated in 100% acetone and ethanol, respectively, at -20 °C. The pellets were resuspended in deglycosylation buffer (0.2 mol/L Tris, 0.2 mol/L sodium acetate, 0.1 mol/L EDTA, 50 mmol/L sodium phosphate, pH=6.8). The following deglycosylation enzymes were used: endo-α-N-acetylgalactosaminidase, β1,4-galactosidase, β-N-acetylglucosaminidase, α-2-3,6,8,9-Neuraminidase (all from Merck-Millipore Glycoprotein Deglycosylation Kit, 362280), Chondroitinase ABC (Sigma-Aldrich, C3667), Heparinase II (Sigma-Aldrich, H6512), and Endo-β1,4-galactosidase (Sigma-Aldrich, G6920). Samples were incubated for 1 h at 25°C, followed by 24 h at 37°C in agitation, then dried using SpeedVac. Subsequently, samples were reconstituted in O^18^ water containing N-Glycosidase F (PNGase F, from the Merck-Millipore Glycoprotein Deglycosylation Kit, 362280) and incubated at 37°C with shaking for 36 h.

Proteins of all three extracts (SDS and deglycosylated GuHCl/NaCl) were denatured using 9 M urea, 3 M thiourea in a 1:2 ratio (final concentration 6 M urea, 2 M thiourea) and reduced by adding 100 mM DTT (final concentration 10 mM) for each sample and incubated for 1 h at 37 °C. The alkylation was done by adding 500 mM iodoacetamide (final concentration 50mM) in the dark for 1 h. Afterwards, samples were precipitated with pre-chilled acetone and incubated O/N in -20 °C. After incubation, samples were centrifuged at 16.000 x g for 40 min at 4 °C and supernatants were removed carefully to avoid pellet disruption. Pellets were further dried in a vacuum concentrator and resuspended in a 40 μl of 0.01 μg/μl trypsin solution (20 µg of trypsin in 2 ml of 0.1 M triethylammonium bicarbonate, pH 8.5 and incubated with gentle mixing at 37 °C for 18 h.

#### Tandem Mass Tag (TMT) labelling for quantitative proteomics

Digested peptides were then labelled using a TMT10 plex Isobaric Mass Tag Labelling Reagent set (Thermo Scientific, 90406) following the manufacturer’s instructions. TMT reagents (5 mg) were dissolved in 256 μl of ACN, and 8.2 μl were added to all peptide samples. After 1 h incubation at RT, the reaction was quenched by adding 1.7 μl of 5 % hydroxylamine.

Labelled samples from each extract were pooled together in a new microcentrifuge tube (i.e., 3 microcentrifuge tubes for 3 extracts) and dried in a vacuum concentrator. Acidification was done using 0.1 % TFA.

Samples were then subjected to High pH Reversed-Phase Peptide Fractionation (Thermo scientific, 84868) which improves the protein sequence coverage and increases the number of identified proteins. Steps were followed as described in the kit user guide. Briefly, labelled peptides were allowed to bind to the hydrophobic resin under aqueous conditions and then were desalted by washing the column with water by low-speed centrifugation. A multi-step gradient of increasing acetonitrile (ACN) concentrations in a volatile high-pH elution solution was then applied to the columns to elute bound peptides into eight different fractions collected by centrifugation. Each fraction was dried in a vacuum centrifuge and resuspended in 50 µl 2 % ACN, 0.05 % TFA (aq). For the SDS samples, the eight fractions were re-combined in three mixtures as described below which allowed for LC-MS/MS analysis using a 250 min runtime method. The NaCl/GuHCl eight fractions underwent LC-MS/MS analysis using a 120 min runtime method.

**Re-combination of SDS fractions for LC-MS/MS analysis.**

| **Mixture 1** | **Mixture 2** | **Mixture 3** |
| --- | --- | --- |
| Fraction 1 | Fraction 2 | Fraction 3 |
| Fraction 4 | Fraction 6 | Fraction 5 |
| Fraction 8 |  | Fraction 7 |

#### Liquid chromatography MS (LC-MS/MS) of TMT-labelled extracts

TMT-labelled, fractionated peptides were analysed using an LC-MS/MS system consisting of a nano-flow UltiMate 3000 high-performance liquid chromatography (HPLC) system (Thermo Scientific) coupled via an EASY-Spray NG Source (Thermo Scientific) to an Orbitrap Fusion Lumos Tribrid MS (Thermo Scientific).

**SDS fractionated extracts:** Peptides were injected onto a C18 cartridge (Thermo Scientific, 160454) at a flow rate of 25 μl/min for 3 min using 0.1 % FA (aq). Peptides were eluted from the trap cartridge and separated using an EASY-Spray C18 column (Thermo Scientific, ES803) at 45 °C and a flow rate of 0.25 μl/min with the following gradient: 0-3 min, 4 % B; 3-10 min, 4-8 % B; 10-200 min, 8-30 % B; 200-210 min, 30-40 % B; 210-215 min, 40-99 % B; 215-220 min, 99 % B; 220-250 min, 4 % B; with A being 0.1 % FA (aq), and B being 80 % ACN, 0.1 % FA (aq).

**NaCl/GuHCl extracts:** Peptides were injected onto a C18 cartridge (Thermo Scientific, 160454) at a flow rate of 25 μl/min for 3 min using 0.1 % FA (aq). Peptides were eluted from the trap cartridge and separated using an EASY-Spray C18 column (Thermo Scientific, ES803) at 45 °C and a flow rate of 0.25 μl/min at the following gradient: 0-10 min, 4-10 % B; 10-75 min, 10-30 % B; 75-80 min, 30-40 % B; 80-85 min, 40-99 % B; 85-89.8 min, 99 % B; 89.8-90 min, 99-4 % B; 90-120 min, 4 % B; with A being 0.1 % FA (aq), and B being 80 % ACN, 0.1 % FA (aq).

MS data were acquired using a Synchronous Precursor Selection (SPS)-MS3 method with a cycle time of 3 s. An Orbitrap full MS scan (scan range 375-1500 m/z; resolution 120,000; max. injection time 50 ms) was followed by a data-dependent MS2 scan using linear ion trap after collision-induced dissociation (CID) fragmentation (MS isolation window 0.7 m/z; CID collision energy 35 %; max. injection time 50 ms), and a data-dependent SPS-MS3 scan using Orbitrap after higher-energy C-trap dissociation (HCD) fragmentation to generate TMT reporter ions from 5 SPS precursors (MS2 isolation window 2 m/z; scan range 100-500 m/z; resolution 60,000; max. injection time 105 ms). Dynamic exclusion was applied to the MS method.

Proteome Discoverer software (Thermo Scientific, version 2.2.0.388) was implemented to search raw data files against the human database (SDS: UniProtKB/Swiss-Prot version from May 2018, 20,349 protein entries; NaCl/GuHCl: UniProtKB/Swiss-Prot version from January 2019, 20,413 protein entries) using Mascot (Matrix Science, version 2.6.0). The mass tolerance was set at 10 ppm for precursor ions and 0.8 Da for fragment ions. Trypsin was set as the protein-digesting enzyme with up to two missed cleavages allowed. Carbamidomethylation of cysteine was chosen as a static modification and, for the SDS extracts, oxidation of methionine was chosen as a dynamic modification. For the NaCl and GuHCl extracts, oxidation of methionine, lysine and proline, and deamidation of asparagine in the presence of ^18^O water were chosen as dynamic modifications. To detect TMT-labelled peptides, the modifications specified in the quantification method (lysine and N-terminal residue modification +229.163 Da) were included in all of the searches. Correction for the reagent lot-specific isotopic impurities of TMT tags was applied (TMT 10plex lot no. TG271817A). To account for variation in amounts between samples, the data were normalised to the total peptide amount. Before exporting the data from Proteome Discoverer for further analysis, it was filtered for Master Proteins, a minimum number of two unique peptides per protein and a High Protein FDR Confidence as determined by Proteome Discoverer’s Protein FDR Validator node (FDR Confidence Threshold for High Confidence = 0.01).

**Supplementary Tables**

**Table S1:** List of core matrisome and matrisome-associated proteins detected by mass spectrometry in NaCl extracts of PLF-LG and NEF-HG patients.

| **Protein name** | **Division** | **Category** | **Gene name** | **Log_2_ FC** | **P value** |
| --- | --- | --- | --- | --- | --- |
| Thrombospondin-4  (TSP-4) | Core matrisome | ECM Glycoproteins | THBS4 | -1.316 | **0.008*** |
| Collagen alpha-1(XII) chain | Core matrisome | Fibril-associated collagens with interrupted triple helices | COL12A1 | -1.114 | **0.009*** |
| Glia-derived nexin | Matrisome-associated | ECM Regulators | SERPINE2 | -0.792 | **0.034*** |
| Host cell factor 1 | Matrisome-associated | Secreted Factors | HCFC1 | 0.377 | **0.042*** |
| Lactadherin | Core matrisome | ECM Glycoproteins | MFGE8 | -0.654 | 0.066 |
| Complement C1q subcomponent subunit B | Matrisome-associated | ECM-affiliated Proteins | C1QB | -0.587 | 0.070 |
| Chondroitin sulfate proteoglycan 4 | Matrisome-associated | ECM-affiliated Proteins | CSPG4 | -0.436 | 0.079 |
| Collagen alpha-1(XXVIII) | Core matrisome | Collagens | COL28A1 | 1.175 | 0.091 |
| Latent-transforming growth factor beta-binding protein 4 | Core matrisome | ECM Glycoproteins | LTBP4 | -0.600 | 0.100 |
| Cathepsin L1 | Matrisome-associated | ECM Regulators | CTSL | 0.381 | 0.125 |
| Fibulin-1 | Core matrisome | ECM Glycoproteins | FBLN1 | -1.007 | 0.126 |
| Biglycan | Core matrisome | Proteoglycans | BGN | -0.660 | 0.136 |
| C-type lectin domain family 14 member A | Matrisome-associated | ECM-affiliated Proteins | CLEC14A | 0.425 | 0.137 |
| Complement C1q subcomponent subunit A | Matrisome-associated | ECM-affiliated Proteins | C1QA | -0.450 | 0.153 |
| Serpin B6 | Matrisome-associated | ECM Regulators | SERPINB6 | -0.237 | 0.163 |
| SPARC-like protein 1 | Core matrisome | ECM Glycoproteins | SPARCL1 | 0.403 | 0.185 |
| EGF-containing fibulin-like extracellular matrix protein 1 | Core matrisome | ECM Glycoproteins | EFEMP1 | -0.661 | 0.193 |
| Corticosteroid-binding globulin | Matrisome-associated | ECM Regulators | SERPINA6 | -0.410 | 0.201 |
| Galectin-3 | Matrisome-associated | ECM-affiliated Proteins | LGALS3 | 0.342 | 0.261 |
| Protein S100-A10 | Matrisome-associated | Secreted Factors | S100A10 | 0.178 | 0.268 |
| Annexin A2 | Matrisome-associated | ECM-affiliated Proteins | ANXA2 | -0.160 | 0.271 |
| Versican core protein | Core matrisome | Proteoglycans | VCAN | -0.703 | 0.279 |
| Cathepsin G | Matrisome-associated | ECM Regulators | CTSG | -0.358 | 0.279 |
| Coagulation factor XIII A chain | Matrisome-associated | ECM Regulators | F13A1 | -0.247 | 0.301 |
| Laminin subunit alpha-4 | Core matrisome | ECM Glycoproteins | LAMA4 | -0.126 | 0.318 |
| Laminin subunit beta-2 | Core matrisome | ECM Glycoproteins | LAMB2 | -0.304 | 0.332 |
| Laminin subunit beta-1 | Core matrisome | ECM Glycoproteins | LAMB1 | -0.183 | 0.377 |
| Collagen alpha-1(III) chain | Core matrisome | Collagens | COL3A1 | -1.565 | 0.400 |
| Antileukoproteinase | Matrisome-associated | ECM Regulators | SLPI | 0.592 | 0.416 |
| Hyaluronan-binding protein 2 | Matrisome-associated | ECM Regulators | HABP2 | 0.213 | 0,492 |
| Collagen alpha-1(I) chain | Core matrisome | Collagens | COL1A1 | -0.266 | 0.512 |
| Alpha-1-antichymotrypsin | Matrisome-associated | ECM Regulators | SERPINA3 | 0.192 | 0.540 |
| Annexin A3 | Matrisome-associated | ECM-affiliated Proteins | ANXA3 | 0.182 | 0.552 |
| Serpin B9 | Matrisome-associated | ECM Regulators | SERPINB9 | -0.096 | 0.591 |
| Agrin | Core matrisome | ECM Glycoproteins | AGRN | -0.153 | 0.617 |
| Serpin H1 | Matrisome-associated | ECM Regulators | SERPINH1 | -0.093 | 0.684 |
| Fibrinogen gamma chain | Core matrisome | ECM Glycoproteins | FGG | -0.072 | 0.739 |
| Inter-alpha-trypsin inhibitor heavy chain H3 | Matrisome-associated | ECM Regulators | ITIH3 | -0.064 | 0.755 |
| Tetranectin | Matrisome-associated | ECM-affiliated Proteins | CLEC3B | -0.139 | 0.799 |
| Annexin A11 | Matrisome-associated | ECM-affiliated Proteins | ANXA1 | -0.330 | 0.134 |
| Insulin-like growth factor-binding protein 7 | Core matrisome | ECM Glycoproteins | IGFBP7 | 0.085 | 0.835 |
| Protein S100-A13 | Matrisome-associated | Secreted Factors | S100A13 | 0.050 | 0.900 |
| Mimecan | Core matrisome | Proteoglycans | OGN | -0.025 | 0.931 |
| Protein S100-A4 | Matrisome-associated | Secreted Factors | S100A4 | 0.003 | 0.990 |

Log_2_ fold change (FC) ratio (PLF-LG/ NEF-HG) was calculated and statistical analysis was carried out using unpaired Student´s t-test. *p <0.05 are labelled in bold.

**Table S2:** List of core matrisome and matrisome-associated proteins detected by mass spectrometry in GuHCl extracts of PLF-LG and NEF-HG patients.

| **Protein name** | **Division** | **Category** | **Gene name** | **Log_2_ FC** | **P value** |
| --- | --- | --- | --- | --- | --- |
| Cystatin-C | Matrisome-associated | ECM Regulators | CST3 | 0.278 | 0.074 |
| Tenascin | Core matrisome, | ECM Glycoproteins | TNC | -1.592 | 0.083 |
| Inter-alpha-trypsin inhibitor heavy chain H2 | Matrisome-associated | ECM Regulators | ITIH2 | -0.652 | 0.092 |
| Alpha-1-antitrypsin | Matrisome-associated | ECM Regulators | SERPINA1 | 0.395 | 0.095 |
| Laminin subunit beta-2 | Core matrisome | ECM Glycoproteins | LAMB2 | 0.356 | 0.099 |
| Lactadherin | Core matrisome | ECM Glycoproteins | MFGE8 | -0.645 | 0.101 |
| Basement membrane-specific heparan sulfate proteoglycan core protein | Core matrisome | Proteoglycans | HSPG2 | 0.331 | 0.112 |
| Versican | Core matrisome | Proteoglycans | VCAN | -1.376 | 0.130 |
| Laminin subunit beta-1 | Core matrisome | ECM Glycoproteins | LAMB1 | 0.385 | 0.140 |
| Collagen alpha-2(V) chain | Core matrisome, | Collagens | COL5A2 | -1.036 | 0.144 |
| Collagen alpha-1(V) chain | Core matrisome | Collagens | COL5A1 | -1.045 | 0.159 |
| Collagen alpha-1(VI) chain | Core matrisome | Collagens | COL6A1 | -0.439 | 0.170 |
| Laminin subunit alpha-2 | Core matrisome | ECM Glycoproteins | LAMA2 | 0.581 | 0.175 |
| Multimerin-2 | Core matrisome | ECM Glycoproteins | MMRN2 | 0.443 | 0.177 |
| Decorin | Core matrisome | Proteoglycans | DCN | -0.900 | 0.179 |
| Inter-alpha-trypsin inhibitor heavy chain H1 | Matrisome-associated | ECM Regulators | ITIH1 | -0.796 | 0.179 |
| Latent-transforming growth factor beta-binding protein 1 | Core matrisome | ECM Glycoproteins | LTBP1 | -0.380 | 0.187 |
| Protein-glutamine gamma-glutamyltransferase 2 | Matrisome-associated | ECM Regulators | TGM2 | -0.548 | 0.203 |
| Collagen alpha-1(I) chain | Core matrisome | Collagens | COL1A1 | -0.950 | 0.209 |
| Collagen alpha-2(I) chain | Core matrisome | Collagens | COL1A2 | -0.882 | 0.214 |
| Cystatin-B | Matrisome-associated | ECM Regulators | CSTB | 0.282 | 0.227 |
| Periostin | Core matrisome | ECM Glycoproteins | POSTN | -1.351 | 0.245 |
| Biglycan | Core matrisome, | Proteoglycans | BGN | -1.078 | 0.275 |
| Fibrillin-1 | Core matrisome | ECM Glycoproteins | FBN1 | -0.645 | 0.314 |
| Protein AMBP | Matrisome-associated | ECM Regulators | AMBP | -0.367 | 0.320 |
| Hyaluronan and proteoglycan link protein 1 | Core matrisome | Proteoglycans | HAPLN1 | -0.608 | 0.340 |
| Cathepsin G | Matrisome-associated | ECM Regulators | CTSG | 0.409 | 0.346 |
| Protein CYR61 | Core matrisome | ECM Glycoproteins | CYR61 | -0.551 | 0.346 |
| Thrombospondin type-1 domain-containing protein 4 | Core matrisome | ECM Glycoproteins | THSD4 | -0.197 | 0.481 |
| Plasminogen | Matrisome-associated | ECM Regulators | PLG | 0.294 | 0.603 |
| Lumican | Core matrisome | Proteoglycans | LUM | -0.273 | 0.634 |
| Alpha-2-macroglobulin | Matrisome-associated | ECM Regulators | A2M | 0.118 | 0.728 |
| Antithrombin-III | Matrisome-associated | ECM Regulators | SERPINC1 | -0.082 | 0.778 |
| Laminin subunit alpha-4 | Core matrisome | ECM Glycoproteins | LAMA4 | 0.032 | 0.819 |
| Fibrinogen beta chain | Fibrinogen beta chain | ECM Glycoproteins | FGB | 0.140 | 0.872 |
| Kininogen-1 | Matrisome-associated | ECM Regulators | KNG1 | -0.029 | 0.876 |
| Collagen alpha-2(IV) chain | Core matrisome | Collagens | COL4A2 | -0.053 | 0.891 |
| Collagen alpha-1(XV) chain | Core matrisome | Collagens | COL15A1 | -0.028 | 0.895 |
| Fibrinogen gamma chain | Core matrisome | ECM Glycoproteins | FGG | -0.084 | 0.905 |
| Fibrinogen alpha chain | Core matrisome | ECM Glycoproteins | FGA | 0.947 | 0.055 |
| Galectin-3 | Matrisome-associated | ECM-affiliated Proteins | LGALS3 | 0.008 | 0.951 |

Log_2_ fold change (FC) ratio (PLF-LG/ NEF-HG) was calculated and statistical analysis was carried out using unpaired Student´s t-test. *p <0.05 are labelled in bold.

**Supplementary Figures**


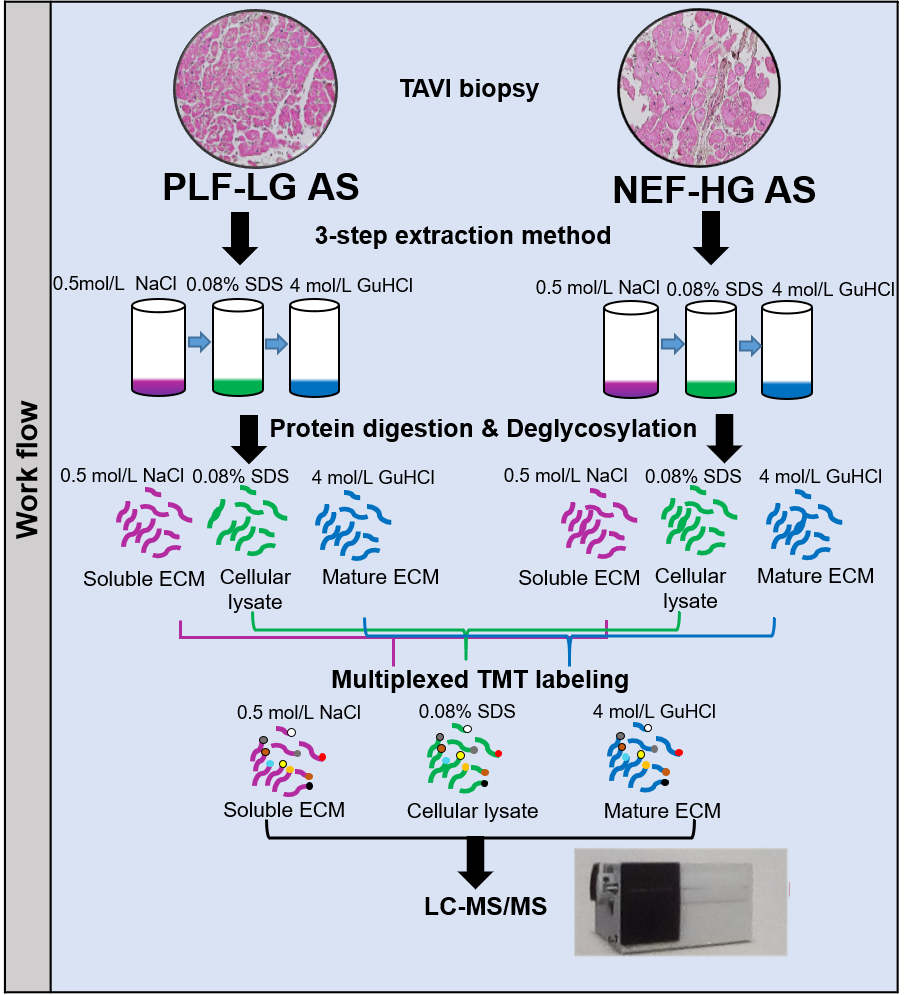


**Figure S1: Extraction of cardiac ECM proteins and quantitative LV proteomics using multiplexed mass spectrometry. LV cardiac biopsies were subjected to the sequential three-step extraction method yielding three different extracts per sample.** NaCl (enriched with newly synthesized and/or loosely bound ECM proteins), SDS (enriched with cellular proteins) and GuHCl (enriched with insoluble, highly integrated ECM proteins such as cross-linked collagens, proteoglycans and glycoproteins). Each extract was labeled with **tandem mass tags (TMT) for quantitative proteomic analyses using** liquid chromatography coupled with tandem mass spectroscopy (LC-MS/MS). **TAVI: transcatheter aortic valve implantation. PLF-LG: paradoxical low-flow low-gradient aortic stenosis, NEF-HG: normal ejection fraction high-gradient aortic stenosis.**


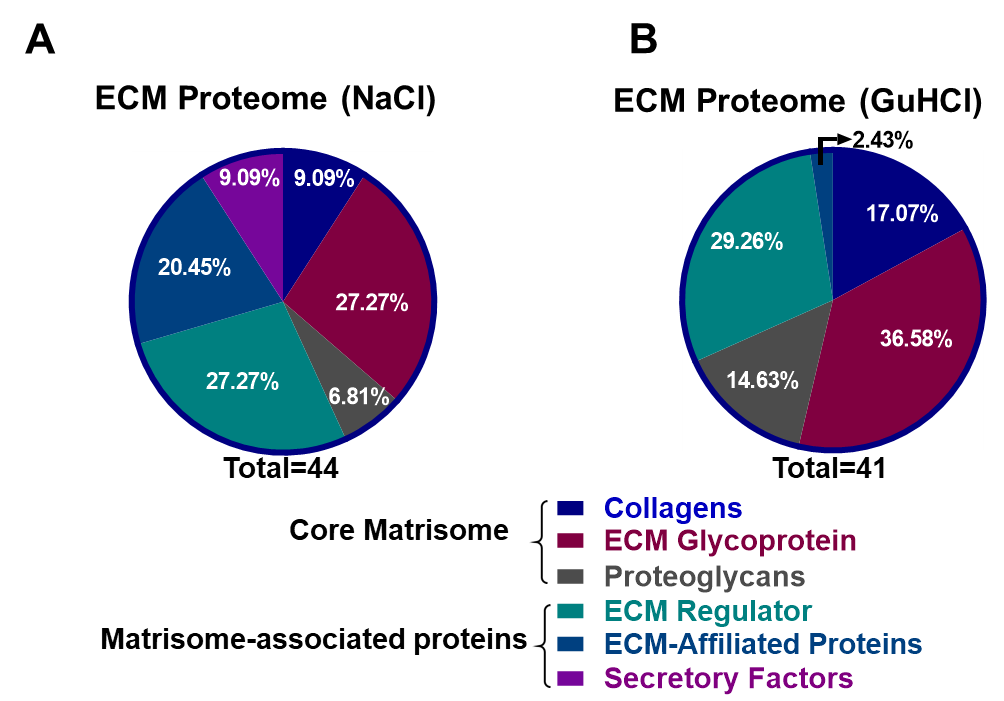


**Figure S2:** **Categorization of extracellular matrix (ECM) proteins. Pie charts show total numbers of identified ECM proteins in the NaCl and the GuHCl extracts grouped by matrisome categories. Two main categories are core matrisome and matrisome-associated proteins. The core matrisome and matrisome-associated proteins are subdivided into three subgroups each (core matrisome: collagens, ECM glycoproteins, and proteoglycans; matrisome-associated proteins: ECM regulators, ECM-affiliated proteins, and secretory factors).**
